# Size fractionated NET-Seq reveals a conserved architecture of transcription units around yeast genes

**DOI:** 10.1002/yea.3931

**Published:** 2024-03-03

**Authors:** Shidong Xi, Tania Nguyen, Struan Murray, Phil Lorenz, Jane Mellor

**Affiliations:** Department of Biochemistry, University of Oxford, Oxford, UK

**Keywords:** gene regulation, nascent transcript mapping, Reb1, *S. cerevisiae*, size fractionated NET-Seq, transcription

## Abstract

Genomes from yeast to humans are subject to pervasive transcription. A single round of pervasive transcription is sufficient to alter local chromatin conformation, nucleosome dynamics and gene expression, but is hard to distinguish from background signals. Size fractionated native elongating transcript sequencing (sfNET-Seq) was developed to precisely map nascent transcripts independent of expression levels. RNAPII-associated nascent transcripts are fractionation into different size ranges before library construction. When anchored to the transcription start sites (TSS) of annotated genes, the combined pattern of the output metagenes gives the expected reference pattern. Bioinformatic pattern matching to the reference pattern identified 9542 transcription units in *Saccharomyces cerevisiae*, of which 47% are coding and 53% are noncoding. In total, 3113 (33%) are unannotated noncoding transcription units. Anchoring all transcription units to the TSS or polyadenylation site (PAS) of annotated genes reveals distinctive architectures of linked pairs of divergent transcripts approximately 200nt apart. The Reb1 transcription factor is enriched 30nt downstream of the PAS only when an upstream (TSS −60nt with respect to PAS) noncoding transcription unit co-occurs with a downstream (TSS +150nt) coding transcription unit and acts to limit levels of upstream antisense transcripts. The potential for extensive transcriptional interference is evident from low abundance unannotated transcription units with variable TSS (median −240nt) initiating within a 500nt window upstream of, and transcribing over, the promoters of protein-coding genes. This study confirms a highly interleaved yeast genome with different types of transcription units altering the chromatin landscape in distinctive ways, with the potential to exert extensive regulatory control.

## 1 Introduction

Eukaryotic genomes from yeast to humans are pervasively transcribed on both strands, upstream, downstream or antisense to regions annotated as genes (Mellor et al., 2016; Murray & Mellor, 2016; Murray et al., 2012, 2015; Nguyen et al., 2014; Pelechano et al., 2013; Thomas et al., 2020) but challenges remain in precisely mapping where each transcription event starts and ends. This is important as barely detectable levels of overlapping transcription can bring about transcriptional interference of nearby genes with a profound impact on the biology of an organism (Berretta et al., 2008; Bierhoff et al., 2010; Brown et al., 2018; Bumgarner et al., 2012; Colin et al., 2011; du Mee et al., 2018; He et al., 2008; Hongay et al., 2006; Katayama et al., 2005; Kuryan et al., 2012; Murray & Mellor, 2016; Murray et al., 2012, 2015; Nguyen et al., 2014; Ni et al., 2010; Pelechano & Steinmetz, 2013; Prescott & Proudfoot, 2002).

Techniques such as transcript isoform sequencing (TIF-Seq) which involves circularisation of individual mature transcripts to precisely map the 5′ (transcription start site, TSS) and 3′ end (polyadenylation site, PAS) have provided a detailed map of the yeast transcriptome (Pelechano et al., 2013, 2014) and the more complex mammalian mature transcriptome (TIF-Seq. 2) (Wang et al., 2020), revealing extensive transcript heterogeneity and bi-cistronic transcripts over tandemly arranged genes. However, TIF-Seq requires an m7G-cap and poly A tail and thus maps fully processed transcripts. Underrepresented transcripts include those that are lowly expressed or rapidly degraded, such as cryptic unstable transcripts (CUTs). To capture such transcripts, techniques to map nascent transcripts can be used, such as native elongating transcript sequencing (NET-Seq) (Churchman & Weissman, 2011; Fischl et al., 2017; Nguyen et al., 2014; Nojima et al., 2015) or PRO-Seq (Booth et al., 2016; Kwak et al., 2013; Mahat et al., 2016). Both techniques capture the nucleotide in the active site of engaged RNAPII and facilitate base-pair resolution mapping of RNAPII occupancy across the genome. In NET-Seq, this includes elongating, paused/stalled or backtracked RNAPII, while PRO-Seq provides complementary information on polymerases that have recently translocated and incorporated a labelled nucleotide during the pulse. Although these methods capture unstable nascent transcripts, it can be difficult to unambiguously distinguish reads from the background signal. Furthermore, the missing information in NET-Seq and PRO-Seq is which transcription unit generates the reported reads.

To distinguish individual transcription units in the nascent transcriptome of *Saccharomyces cerevisiae*, size fractionated NET-Seq (sfNET-Seq) was developed. Nascent RNA from immunoprecipitated RNAPII was isolated, a linker ligated to uniquely mark the nascent 3′ end, PAGE gel electrophoresis used to split the nascent RNA into several size fractions and libraries prepared with each fraction. Together, these libraries yielded a nested set of output reads that together allow the unambiguous and precise definition of individual transcription unit. By pattern matching processed sfNET-Seq data at each position across the genome to a reference pattern derived using well-annotated transcription units, a total of 9542 transcription unit, of which 3113 (33%) are newly identified unannotated noncoding transcription units, can be unambiguously defined. sfNET-Seq allows clear distinction of overlapping transcription units that cannot be separated using conventional NET-Seq (Churchman & Weissman, 2011; Fischl et al., 2017) or PRO-Seq (Booth et al., 2016). The potential for extensive transcriptional interference is evident from the overlapping transcription units and from newly mapped low abundance unannotated transcription units with variable TSS (median −240nt) initiating within a 500nt window upstream of, and transcribing over, the promoters of protein-coding genes. By anchoring all transcription unit discovered using sfNET-Seq to the reference set of protein-coding genes, a distinctive architecture of linked pairs of divergent transcription units approximately 200nt apart at both the TSS and PAS was uncovered. At the PAS, the Reb1 transcription factor is enriched 30nt downstream only when an upstream (TSS −60nt) noncoding transcription unit co-occurs with a downstream (TSS +150nt) coding transcription unit. In the context of divergent coding and noncoding transcription units, engineering and ablating a Reb1 transcription factor-binding site leads to an increase in Rrp6-dependent noncoding transcripts but no change in levels of the divergent coding transcripts. This refines the context of genome-wide Reb1 function and confirms a highly interleaved yeast genome with differentially regulated transcription units altering the chromatin landscape in distinctive ways and with the potential to exert extensive regulatory control. Finally, this high-resolution data set allows the accurate characterisation of regulatory regions and chromatin environment, and uncovers new relationships between the nascent transcriptome and protein-coding genes.

## 2 Material and Methods

### 2.1 Size fractionated NET-Seq

Size fractionated NET-Seq was developed to map new transcription units and their putative TSS on the *S. cerevisiae* (BY4741) genome. The steps up to ligation of linker-1 were performed as for the conventional NET-Seq protocol (Fischl et al., 2017; Uzun et al., 2021). For size fractionated NET-Seq, 6 μg of RNA (>3 μg) was used and split into six non-stick RNase-free tubes for the ligation reaction, just as for conventional NET-Seq. After 3 h incubation at 37°C, the reaction was quenched by adding 0.7 μL of 0.5 M EDTA to each tube. The ligation product was purified and concentrated by isopropanol precipitation. Specifically, 0.56 mL of RNA solution and 0.75 mL of isopropanol were added to each tube. After vortexing, the mixture was incubated at −20°C for 2 h, followed by centrifugation at 4°C, 20,000*g* for 30 min. Next, the supernatant was removed without disturbing the RNA pellet. The pellet was then washed once with 0.75 mL of 80% ice-cold ethanol. After brief centrifugation, residual ethanol was carefully removed by pipetting. The pellet was air-dried at room temperature for 10 min. The RNA pellets were resuspended in 15 μL of 10 mM Tris.Cl (pH 7.0), mixed with 15 μL of 2× TBU denaturing loading buffer, denatured at 80°C for 3 min, and 10 μL loaded onto three wells of an ice-cold 3.5% TBU polyacrylamide gel (10 mL 4.8 g Urea, 1.17 mL 30% acrylamide solution, 50 μL 10% APS, 1 mL 10× UltraPure TBE buffer, MilliQ water to 10 mL, 10 μL TEMED) poured between glass plates cleaned with 1% SDS and washed thoroughly with MilliQ water to remove RNase contamination. The gels were left for 1 h to set and then pre-run for 15 min. Before loading, a pipette with a clean tip was used to flush the wells with 1× TBE buffer several times. Two empty wells were left between the ladders (heated denatured 10, 50, 100, and 1 kb DNA ladders) and the sample. The samples were subject to electrophoresis using ice-cold 1× TBE running buffer, at 80 V for 80 min. The gel was stained for 5 min in 50 mL of 1× TBE buffer containing 5 μL of SYBR Gold nucleic acid gel stain (S11494, Thermo Fisher Scientific), followed by destaining in 1× TBE buffer for another 5 min. The gel was frozen at −20°C for 15 min, before cutting into size ranges, normally 20–50nts, 50–170nts, 170–500nts and 500–1000nts. The RNA was recovered from mashed gel pieces using electroelution in 1× TBE at 80 V with the electrophoresis tank sitting in wet ice. Fluorescence was monitored every 10 min with the transilluminator, with 10 min being sufficient for the smallest fraction and up to 2 h for the longest RNA. After electroelution, RNA from each size fraction was dissolved in 600 μL of 10 mM Tris. Cl (pH 7.0). To purify and concentrate the RNA, 2 μL of Glycoblue, 50 μL of 3 M sodium acetate (pH 5.5) and 0.75 mL of isopropanol were added. The RNA pellet of the smallest fraction was dissolved in 10 μL of 10 mM Tris.Cl (pH 7.0). All the other size fractions were dissolved in 20 μL of the same buffer. The RNA from each fraction, except the smallest, is subject to alkaline fragmentation and library construction, with *S. pombe* NET-seq spike-in, following the conventional NET-Seq protocol (Fischl et al., 2017; Uzun et al., 2021), except that up to two more PCR cycles may be needed. The sequencing of NET-Seq libraries was performed on the Illumina NEXTSeq.500 Sequencer with the high throughput flow cell, single-end reads and 50 cycles. NET-Seq normally requires 100 million reads to obtain good coverage on each *S. pombe*-spiked *S. cerevisiae* sample.

### 2.2 Bioinformatics analysis of size-selected NET-Seq output

The adapter sequence ‘ATCTCGTATGCCGTCTTCTGCTTG’ was first trimmed from the raw reads by running cutadapt. Bowtie was utilised to align the reads to the hybrid genome of *S. pombe* and *S. cerevisiae* with the following settings: ‘-q -p 16 -S -m 1 -n 1 -e 70 -l 28 -k 1 -- best -- phred33-quals’. The uniquely aligned sam files were converted into the bedgraph files by samtools, bamtobed and genomecov. The data are aligned as for conventional NET-Seq (Churchman & Weissman, 2011; Fischl et al., 2017). For the smallest size fraction (20–50nt), the reads trimming by ‘genomecov’ was performed on either the 3′ ends or the 5′ ends of reads, which produced two sets of bedgraph files 5′ and 3′. Bedgraph files were visualised using IGV (Thorvaldsdottir et al., 2013). The bedgraph files produced from each size fraction of nascent RNA were imported into MATLAB and smoothed by zero-phase digital filtering algorithm ‘filtfilt’. Specifically, the digital filter was first set up by using function ‘designfilt’ with the following parameters ‘lowpassiir’, ‘Filter-Order’, 12, ‘HalfPowerFrequency’, 0.05, ‘DesignMethod’, ‘butter’. It returned a digital filter object, which was used as one of the inputs of the ‘filtfilt’ function together with the data on each of the size fractionated fractions. The output data were the smoothed data for each of the size fractions. TIF-Seq annotations (Pelechano et al., 2013, 2014) were then utilised to generate a metagene for each of the smoothed size fractions. The data of the first 400nts downstream of the TSS of the metagenes were combined, which was the reference pattern. At each nucleotide position on the genome, the data in the 400nts downstream of that position for all four data tracks were combined, which was the pattern to be tested. The Pearson correlation coefficient was calculated by using the MATLAB function ‘corr’ between the reference pattern and the pattern to be tested. As a result, all positions on the genome had a Pearson correlation coefficient, which was plotted as a curve. The *x*-axis of the curve was the position on the genome. The position of the peaks on the curve was called by using ‘findpeaks’ with the minimal peak height of 0.34 and the minimal peak distance of 50nts. The positions of the peaks were the positions of the putative TSS. A putative TSS was filtered out of the data if the total read number in its downstream 100nt region of all four data tracks was lower than 10. The mapped TSS was compared to several existing annotations, including TIF-Seq and PRO-CAP. A TSS was assigned to an existing annotation if their distance was smaller than 70 bps. The Kolmogorov–Smirnov test and the single-sided Fisher’s test were carried out by using the MATLAB functions ‘kstest2’ and ‘fishertest’, respectively. Metagenes were plotted by following the procedures described by Churchman and Weissman (2011) and Fischl et al. (2017).

### 2.3 Motif and factor enrichment analysis

The motif enrichment analysis was performed on the MEME server version 5.01. Specifically, the differential enrichment mode was used. The size distribution was set as ‘zero or one occurrence per Sequence’. The number of motifs to find was set at 5. ‘0-order model of Sequences’ was chosen as the background model. The width of motifs was set between 6 and 50 bps. Tick the box for searching given strand only but do not tick the box for searching palindromes only. The ChIP-Seq data for Reb1 was retrieved from the GEO database of NCBI, accession number: GSM2143115 (Bosio et al., 2017).

### 2.4 RNA extraction, Northern blots and strand-specific probes

RNA extraction, Northern blots and strand-specific probes were done precisely as described in Nguyen et al. (2014). All experiments were performed at least in duplicate to ensure that the trends observed were reproducible. Briefly, unless otherwise stated, cells were grown to exponential phase in rich medium (YP) supplemented with 2% glucose (YPD). Total RNA extracts were acquired by hot phenol extraction. The concentration of RNA extract was measured and standardised to 1 μg/μL using a nanodrop spectrophotometer. Total RNA samples (~10–20 μg) were separated by electrophoresis on formaldehyde 1% agarose gels containing ethidium bromide (EtBr) for 3 or 6 h at 100 V. To check for RNA degradation, the 25S and 18S ribosomal RNA (rRNA) bands were photographed under UV light using a 0.5 or 0.1 s exposure. These images also served as loading controls. In vitro transcription with T7 RNA polymerase and radiolabelling was used to create strand-specific probes for Northern blot. DNA templates incorporating a unidirectional T7 phage promoter sequence on one strand were generated by PCR. For each targeted region, two ~200–300 bp templates, a ‘sense’ and an ‘antisense’ were created, differing only in the location of the T7 sequence. With these templates, single-stranded, radiolabelled RNA probes were synthesised through in vitro transcription using a T7-promoter-dependent RNA polymerase (Ambion) in the presence of ^32^P_α_-UTP. The location of the T7 promoter sequence in the template DNA imparted directionality to the reaction, therefore generating strand-specific probes. Strand specificity was confirmed by hybridisation to unlabelled sequences on dot blots. To increase hybridisation specificity and reduce background signal, RNA probes were treated with DNase-I to degrade residual dsDNA, and filtered through a G-350 Sepharose and glass filter column to remove excess nucleotide triphosphates.

### 2.5 p*TEF* deletions and sequence scrambles

Methods to create mutations in Reb1-binding sites in the p*TEF* are done as described previously (Nguyen et al., 2014). Altered sequences are shown in Figure 7.

## 3 Results

### 3.1 Size fractionated NET-Seq, a new method for mapping transcription units

**Figure 1:**
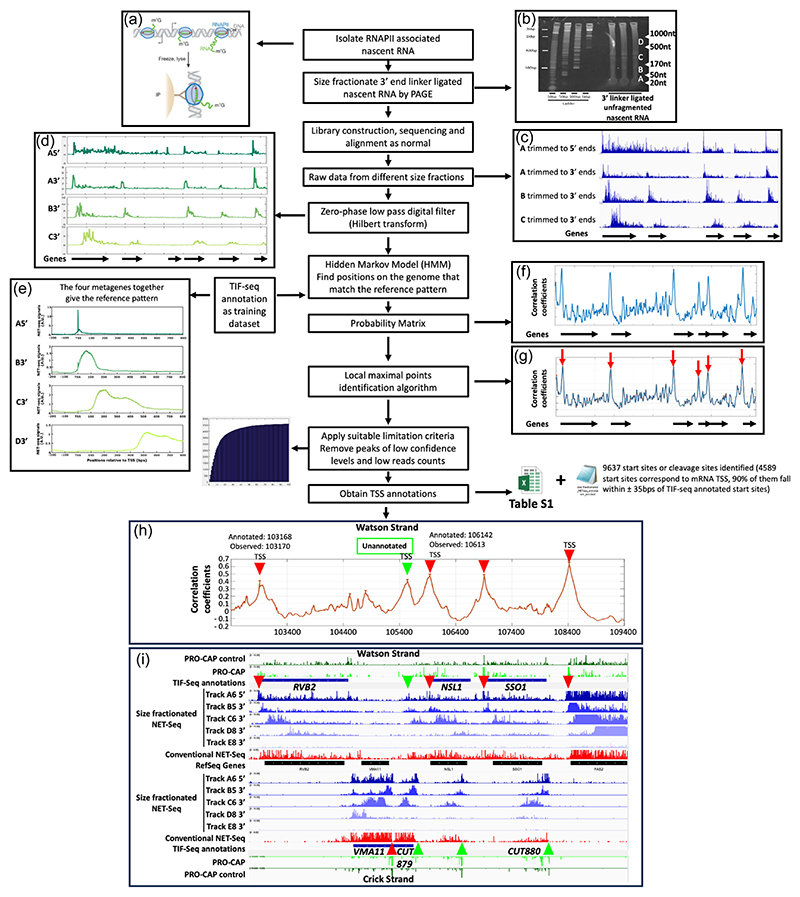
The workflow of the size fractionated NET-seq. (a) After immunoprecipitation of RNAPII, the linker is ligated to the 3′OH of the isolated nascent transcripts. (b) The ligated nascent transcripts of different sizes are separated by polyacrylamide gel electrophoresis and RNA fractions of different size ranges isolated from the gel. (c) The sequencing data from the different size fractioned libraries are aligned and visualised in IGV. Libraries A6 (20–50nts) aligned to the 5′ end and 3′ ends, B5 (50–170nts) aligned to the 3′ end and C6 (170–500nts) aligned to the 3′ are shown. (d) A low-pass digital feature is used to smooth the data visualised for the same region. (e) Transcription start sites (TSS) annotations from TIF-seq (Pelechano et al., 2013) are utilised to plot the metagenes for each library, including D8 (500–1000nts), for known TSS and transcription units in the sfNET-Seq data, which constitute the reference pattern. (f) The correlation coefficients for each genome position yield a smooth curve with peaks, shown for the same region. The positions of the peaks are the genomic positions of TSS. (g) Peak calling algorithms are applied to find the peaks (red arrows), which gives the positions of both previously identified and newly identified TSS and transcription units (see Table S1 for complete data set). (h) An example of pattern matching to identify new transcription units yielding a correlation plot showing the degree of match between the reference pattern and the distribution of reads on the Watson strand of a region of chromosome XVI. The correlation plot peaks around the TSS for the four annotated genes (*YPL235W RVB2, YPL233W NSL1, YPL232W SSO1* and *FAS2)* are indicated with red arrowheads. The TSS for an unannotated transcription unit transcribed into the promoter of *NSL1 (YPL233W)* is indicated with a green arrowhead. (i) IGV snapshot of both strands of the same region of chromosome XVI showing the sfNET-Seq reads (blue), conventional NET-Seq reads (red), Ref-Seq annotations, TIF-Seq annotations (Pelechano et al., 2013, 2014) and PRO-cap annotations (bright green) and controls (dark green) from (Booth et al., 2016). The TSS for coding transcription units are indicated with red arrowheads and noncoding transcription units are indicated with green arrowheads, including *CUT879* transcribed into the promoter of *VMA11* (*YPL234C*), *CUT880* antisense to *SSO1* and unannotated transcription units into the promoter of, and antisense to, *NSL1 (YPL233W)*.

The principle behind size fractionated (sf) NET-Seq is to create libraries to map the 3′ nucleotide in the active site of RNAPII in native nascent transcripts of different length, after immunoprecipitation with RNAPII, and to explore the pattern and distribution of the reads over the genome (see Figure 1a–i). As sfNET-Seq uses nascent RNA as the input material together with a size selection step, it has the potential to map new transcription units and their TSS with greater confidence than other methods which rely on finding single peaks higher than the background threshold in a single library. This applies to PRO-CAP, which is used to annotate TSS (Booth et al., 2016) (Figure 1i), and conventional NET-Seq, where the background signal from an untagged strain is subtracted from the signal obtained with tagged and immunoprecipitated RNAPII (Fischl et al., 2017), as done for the data presented in this study (Figure 1i) or where no background signal control is used (Churchman & Weissman, 2011). In addition, the extent of unstable nascent transcription units can be mapped with confidence and without the use of mutants that knock out parts of the transcript degradation machinery, and lead to a stress response (O'Duibhir et al., 2014), thus distorting the signal intensities. Finally, the method can potentially distinguish the individual nascent transcripts transcribed from overlapping transcription units which cannot be distinguished with convention NET-Seq (Nguyen et al., 2014), TT-Seq (Miller et al., 2011) or PRO-Seq (Booth et al., 2016). Thus, sfNET-Seq should allow lowly expressed transcription units to be detected with high sensitivity and confidence, as the method looks for reads that follow a distinct pattern or distribution.

### 3.2 The workflow of size fractionated NET-Seq for annotating transcription units

The workflow of size fractionated NET-Seq is shown in Figure 1. Nascent RNA was first isolated from the cell lysate using the conventional NET-Seq protocol (Fischl et al., 2017; Uzun et al., 2021), in which nascent transcripts are immunoprecipitated with RNAPII from cell lysates (Figure 1a). The 3′ ends of the nascent RNA molecules were immediately ligated with the 3′ adapter, which marks the original 3′ ends of nascent RNA. Using polyacrylamide gel electrophoresis, the 3′ adapter ligated nascent RNA molecules were split into fractions with different size ranges (Figure 1b). With the exception of the smallest fraction (A; 20–50nt), the eluted nascent transcripts were subject to alkaline fragmentation and then used to create NET-Seq libraries. The barcoded libraries were pooled and sequenced on an Illumina platform. The sequencing reads were first processed by the conventional NET-Seq data processing protocols (Churchman & Weissman, 2011) and visualised using IGV (Thorvaldsdottir et al., 2013) (Figure 1c). The reads from the shortest fraction (A; 20-50nt) were trimmed to either 5′ ends or 3′ ends, which generated two data tracks, A65′ or A63′, respectively. The reads from the remaining libraries 50–170nt, 170–500nt and 500–1000nt were trimmed to 3′ ends, which generated the corresponding data tracks B6, C6 and D8. Data track E8 shows the background signal mainly from rRNA reads that also map to other regions of the genome. The 20–50nt fraction contained RNA that was shorter than the maximal read length of the Illumina Sequencer, peaks in track A should directly map to the TSS region of the transcription unit, particularly A65′, bearing in mind that the first read will be 18–20nt downstream. In data tracks B5, C6 and D8, a cluster of NET-Seq signals across the transcription unit can be seen different distances downstream on each putative TSS (Figure 1c).

To analyse the outputs from the sequencing, each data track was smoothed by applying a low-pass digital filter. This removes spikes in the data but retains the general shapes of the peaks, and can reduce artefacts (Figure 1d). A metagene was first plotted by using the existing TSS annotations created by TIF-Seq (Pelechano et al., 2013). If a certain nucleotide position on the genome was a TSS for a transcription unit, the reference pattern was expected to be seen downstream. The metagenes together gave the reference pattern expected for a TSS followed by a transcription unit (Figure 1e).

These reference metagenes were then used as the basis to search for new TSSs and transcription unit on the genome. sfNET-Seq signals of each nucleotide position were compared to the reference pattern by calculating the Pearson correlation coefficient for each nucleotide position on the genome. When all the Pearson correlation coefficients were plotted, a smooth curve should be seen (Figure 1f). The TSS and associated transcription unit can be mapped by finding the peaks of this smooth curve (Figure 1g). Importantly, using this method, the heights of the peaks were largely independent of the expression levels. Thresholds were applied to balance the sensitivity and specificity of TSS/transcription unit calling, using a false discovery rate of 10^−3^; one false positive was expected in every 1000 mapped TSS/transcription units. The 9637 TSS annotations obtained using sfNET-Seq are shown in Table S1 and illustrated over a region of chromosome XVI (Figure 1h, i). Peak annotations for coding and noncoding TSSs on the Watson strand are shown using red and green arrowheads respectively (Figure 1h). The effectiveness of sfNET-Seq in discovering unannotated transcription units, for example upstream and antisense to *NSL1*, is illustrated (Figure 1i).

### 3.3 Identification of unannotated transcription units using sfNET-Seq

**Figure 2:**
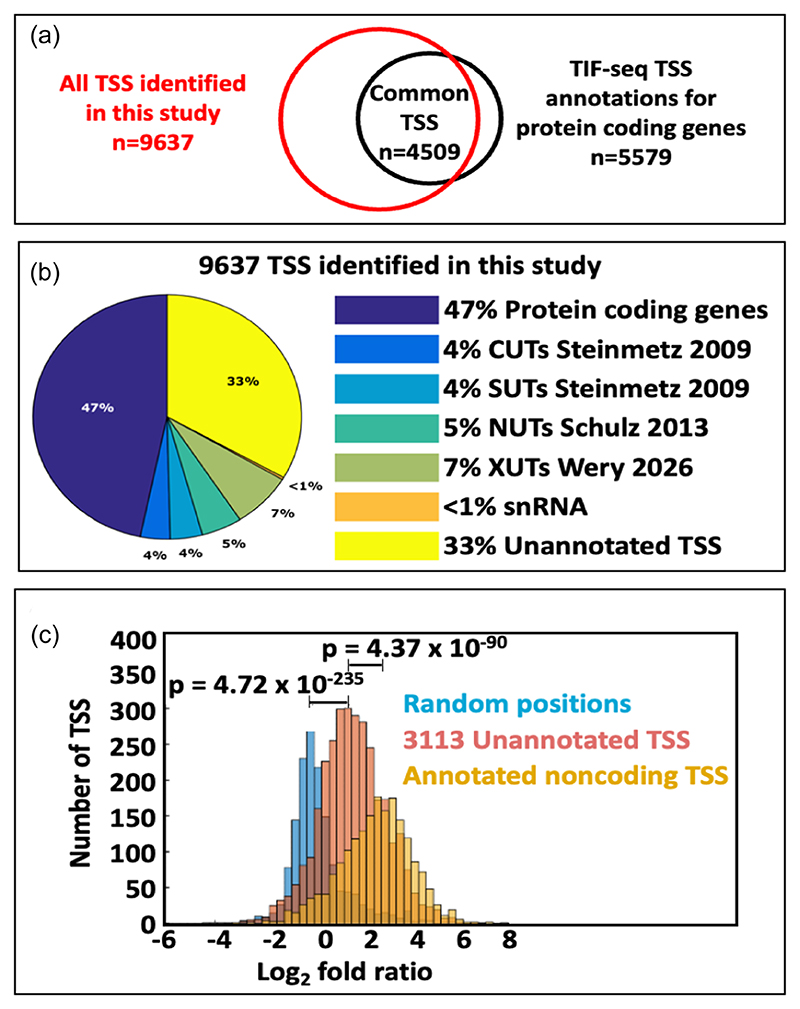
The identities of transcription start sites (TSS) and transcription units mapped by the size fractionated NET-seq. (a) The Venn diagram shows the degree of overlap between the TSS identified by size fractionated NET-seq and the TSS found by TIF-seq (Pelechano et al., 2013). (b) The pie chart reveals the composition of the TSS associated with different types of transcription units. Thirty-three per cent of the 9637 TSS and transcription units found by size fractionated NET-seq are not annotated. (c) Histograms of the log_2_fold ratios between the reads downstream and upstream of the TSS. The annotated noncoding TSS and the unannotated noncoding TSS (identified in this study) are coloured yellow and red, respectively. For comparison, the distribution for random genomic positions is plotted in blue. The Kolmogorov–Smirnov test is used to check whether two distributions are significantly different. The *p* values are marked between the corresponding distributions.

The extent of annotated and unannotated transcription units throughout the yeast genome was explored, as their prevalence can now be unambiguously assigned by sfNET-Seq. To validate the identities of the 9637 putative transcription units from log phase cells (Table S1), they were compared to existing TSS annotations from TIF-Seq (Pelechano et al., 2013). Specifically, if a putative TSS fell within a ±70 bps window of its closest existing annotation, the putative TSS was assigned the identity of that existing annotation. Of the 9637 TSS detected here, 6430 have been annotated in the TIF-Seq data. In all, 4509 of the annotated TSS are associated with protein-coding genes (Cakiroglu et al., 2016) (Figure 2a), while 1921 are of annotated noncoding transcripts, including 344 CUTs (cryptic unstable transcripts; 45% of the 751 currently annotated), 374 SUTs (stable uncharacterised transcripts; 45% of the 794 currently annotated), 704 XUTs (Xrn1-sensitive unstable transcripts), 470 NUTs (Nrd1-dependent unterminated transcript), and 29 snRNA (Pelechano et al., 2013; Wery et al. 2016; Schulz et al. 2013; Xu et al. 2009) (Figure 2b). As many transcripts are targeted by more than one of the nuclear and/or cytoplasmic degradation machineries (e.g., *SUT650* is also annotated as a CUT and a XUT), it is challenging to estimate the proportions of each class detected by sfNET-Seq. Nevertheless, 33% (3208) of the TSS found by size fractionated NET-Seq are not annotated (Figure 2b).

The annotation accuracy was examined by calculating the relative distances between the TSS positions determined using sfNET-Seq and the existing annotations. The deviations in position are plotted as histograms for various types of transcription units (Figure S1). Generally, all histograms have a unimodal distribution centred near the zero point and indicate no obvious systematic error for the TSS positions determined by sfNET-Seq for any type of annotated transcript. As an additional confirmation of the validity of the unannotated transcription units, the reads from a conventional normalised and background-corrected NET-Seq library were counted on either side of the putative TSS of each. The expectation is that more reads would be found downstream from a TSS. The log-transformed ratios of downstream to upstream reads for all the unannotated transcription units are plotted as a histogram in Figure 2c. Although the ratios for the unannotated transcription units are not as high as the ratios for annotated noncoding transcription units, they are significantly higher than the ratios for random positions on the genome (*p* = 4.72 × 10^−235^).

Co-transcriptional cleavage sites in nascent transcripts, such as the polyadenylation site (PAS) or splicing sites, could also yield RNAPII-associated nascent transcript fragments. Of the 3208 unannotated TSS, only 68 and 27 of them can be assigned to known PAS and splicing sites, respectively, and these were removed from subsequent analyses. There remains the possibility that there are unannotated cleavage sites or 5′ ends with RNAPII-associated transcripts resulting from the co-transcriptional action of endo and exonucleases. Detailed analysis of the no-tag controls and pull downs for Rbp3-associated nascent RNA in conventional NET-Seq (Fischl et al., 2017) suggests there is a background signal of short (20–25nt) RNA fragments. As these are evenly distributed across transcription units and not enriched for particular regions of transcription units, they are likely to arise due to the combined action of endo and exonucleases. Evidence for such products is present in the 20–50nt tracks (A libraries) showing a higher background of reads across the transcription unit. Similar small fragments are present in metabolically pulse-labelled nascent transcripts from human cells when subject to size fractionation (Lorenz et al., 2021). As they are restricted to the 20–50nt fraction, these small nascent fragments will not interfere with the annotation of novel transcription units.

### 3.4 Validation of a novel unannotated transcription unit discovered using sfNET-Seq

**Figure 3:**
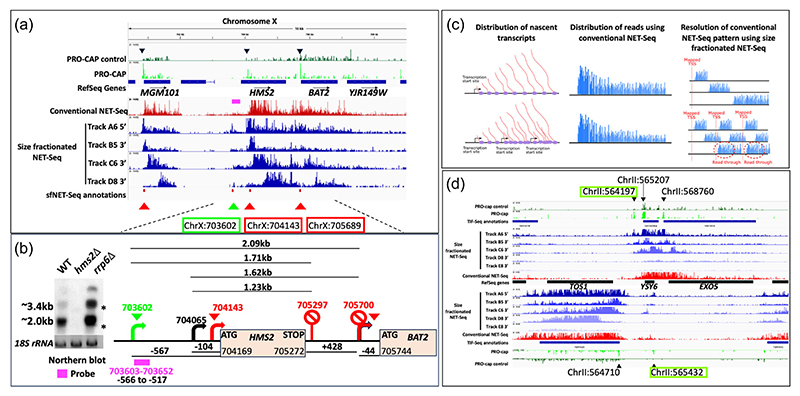
Size fractionated NET-seq can identify novel transcripts and resolve individual transcription units from overlapping transcription at tandemly arranged genes. (a) An IGV snapshot of tandemly arranged genes (*HMS2, BAT2*, and *YJR149W* on the Watson strand of Chromosome X) with overlapping transcription evident from conventional NET-Seq profile (red histograms) and resolution of individual transcription units using size fractionated NET-Seq (blue histograms) showing track A6 (20–50nt aligned to 5′ end), track B5 (50–170nt aligned to 3′ end), track C6 (170–500nt aligned to 3′ end), and track D8 (500–1000nt aligned to 3′ end). Also shown are Ref-Seq annotations and PRO-cap annotations (bright green) and controls (dark green) from Booth et al. (2016). The TSS for coding transcription units are indicated with red arrowheads and for an unannotated noncoding transcription unit with a green arrow head. sfNET-Seq annotations (Table S1) are shown by red ticks and precise TSS positions mapped by sfNET-Seq are shown as numbers in coloured rectangles. (b) Validation of the unannotated transcript initiating at 703602 by sfNET-Seq using a Northern blot (left panel) and strand-specific probe H1 (pink rectangle) taken from (Nguyen et al., 2014). * indicate the position of the 25S and 18S rRNA transcripts. The map of the position of the probe relative to the main TSS of *HMS2* taken from Granovskaia et al. (2010) (black arrow), sfNET-Seq (red arrow) and transcription termination sites (stop signs) taken from Granovskaia et al. (2010) (705297), Nguyen et al. (2014) (705700) and Pelechano et al. (2013, 2014). (c) Schematic representation of the distribution of nascent transcripts, the distribution of read using conventional NET-Seq and the resolution of overlapping transcription unit using size fractionated NET-Seq for a single transcription unit (top) and three tandem transcription units with overlapping transcription (bottom). (d) An IGV snapshot tandemly arranged genes (*YSY6, EXO5*) on Chromosome II with overlapping transcription evident from conventional NET-Seq profile (red histograms) and resolution of individual transcription units, including unannotated transcription units upstream of and antisense to *YSY6* using size fractionated NET-Seq (blue histograms). Other tracks are as in panel a.

An unannotated transcription unit discovered using sfNET-Seq (ChrX:704143; green arrowhead; Figure 3a) was chosen to validate the technique by showing the existence of a distinct transcript using a Northern blot and a strand-specific probe (Figure 3b). This unannotated transcription unit was chosen as it lies upstream of a previously well-characterised cluster of tandemly arranged genes *YJR147W(HMS2)->YJR148W(BAT2)->YJR149W* (Granovskaia et al., 2010; Nguyen et al., 2014; Pelechano et al., 2013). The cluster produces mono-cistronic, bi-cistronic, and tri-cistronic transcripts with alternative polyadenylation sites (~ChrX:705297 and 705700) yielding transcripts >1.2 kb and >1.625 initiating from the *HMS2* TSS (Nguyen et al., 2014). The position of the mapped TSS for *HMS2* (ChrX:704065) from a tiling array (Granovskaia et al., 2010) corresponds well with the sfNET-Seq annotation (ChrX:704143; Table S1, red arrowhead Figure 3).

sfNET-Seq maps the TSS for the unannotated transcription unit at ~ChrX:703602 (green arrowhead; Table S1 and Figure 3a,b), initiating ~540nt upstream of the TSS for *HMS2*. A strand-specific probe (pink rectangle; ChrX:703603-703652), located upstream of the most proximal annotated *HMS2* TSS, hybridises to a transcript larger (~2.0 kb) than the 18S rRNA marker (1789nt), consistent with transcripts (>2.093 and >1.71 kb) extending to the PAS of *HMS2* (Figure 3b). In addition, longer read-through transcripts are evident, consistent with transcription extending to the end of *BAT2* and into *YJR149W* but subject to degradation by Rrp6 in the nuclear exosome.

### 3.5 sfNET-Seq can identify distinct transcription units that are convoluted in conventional NET-Seq

In addition to identifying previously uncharacterised transcripts and transcription units in the yeast genome, the capacity of sfNET-Seq to distinguish individual transcription units within regions of overlapping transcription was examined in more detail. Tandemly arranged genes in the yeast genome often show overlapping transcription, which cannot be resolved using conventional NET-Seq (Figure 3c). Loci with tandemly arranged genes and overlapping transcription producing bi-cistronic transcripts identified in TIF-Seq (Pelechano et al., 2013) were examined to ask if sfNET-Seq could distinguish the individual transcription units (e.g., *NSL1*-SSO1 (Figure 1i), *HMS2->BAT2* (Figure 3a), *YSY6->EXO5* (Figure 3d), *PCL7->DFG10; LYS9->MSO1; ACF4->EAF6* (Figure S2).

Unlike the conventional NET-Seq reads (red track), sfNET-Seq (blue tracks) is able to resolve each coding transcription unit (red arrowheads), and a number of existing and additional new nascent transcripts including extensive antisense transcripts around each of these loci (green arrowheads), with confidence, as each produces the distinct pattern expected by the sfNET-Seq algorithm. In addition, many of the unannotated transcription units also have PRO-CAP signals (bright green tracks), illustrating the utility of sfNET-Seq to identify novel transcription units. PRO-CAP peaks for the unannotated upstream transcript, the main TSS for *YBR162W->A*, the unannotated transcript antisense to *YBR162W->A* and the downstream gene arranged in tandem (*YBR163W; EXO5*) are evident validating their identity as bone fide transcription units (Figure 3d). Thus, size fractionated NET-Seq identifies TSS and their associated transcription units specifically and most of the remaining 3103 unannotated TSS are unlikely to be random artefacts. Examination of the IGV snapshots indicated that many annotated noncoding transcription units (generally CUTs, SUTs, XUTs or NUTs) are found antisense to the protein-coding genes arranged in tandem and there are many examples of noncoding annotated and unannotated transcripts initiating upstream of the main TSS. This prompted a more detailed analysis of the relative positions of these high levels of pervasive transcription throughout the yeast genome.

### 3.6 The precise distribution of coding and noncoding transcription units around protein-coding genes can be revealed by size fractionated NET-Seq

**Figure 4:**
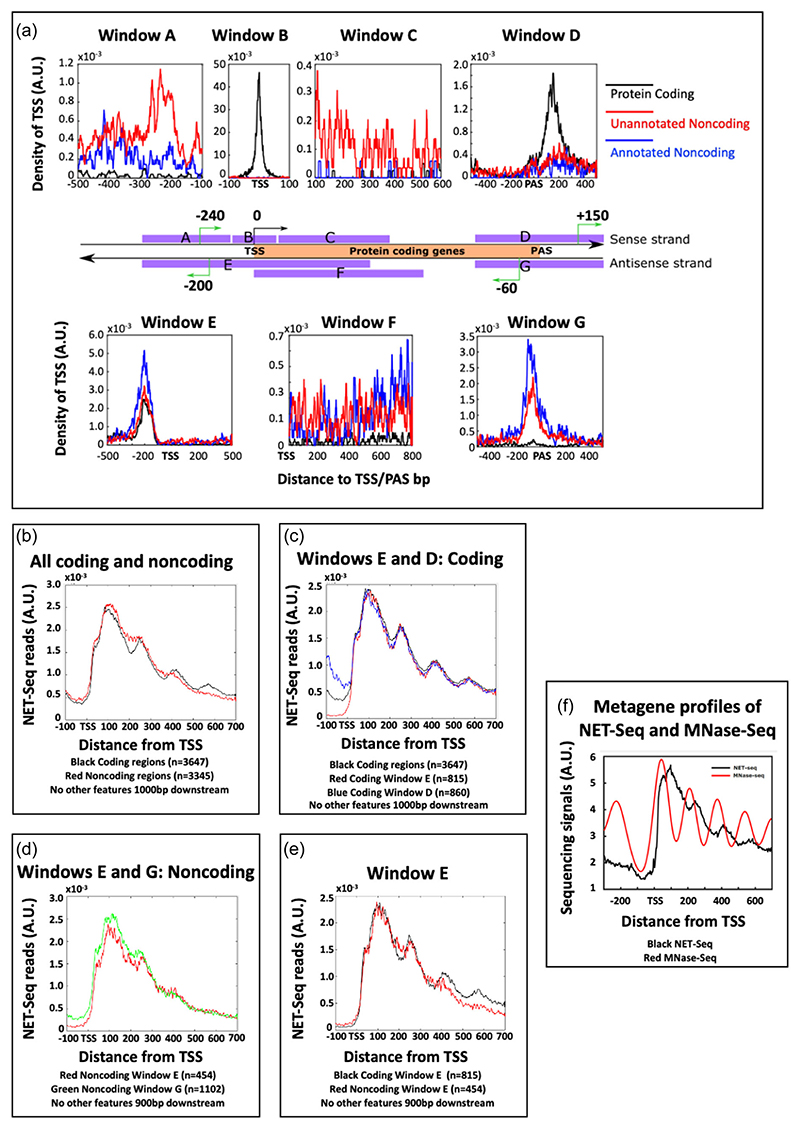
The distributions of transcription start sites (TSS) and transcription units around 5579 protein-coding genes. (a) The start sites of transcription units were mapped both upstream and downstream of TSS and polyadenylation site (PAS) of protein-coding genes on both strands, which produces the seven regions from A to G. The coverage of each region relative to the protein-coding gene metagene (orange box) is represented with the purple bars. The frequently found TSS positions relative to the protein-coding gene metagene are represented with green arrows. For each plot, the *y*-axis is the density of TSS mapped to each nucleotide position, while the *x*-axis is the relative distance to TSS or PAS. The distances for downstream and upstream positions are denoted with positive and negative values, respectively. (b–e) Metagenes of NET-Seq reads for each type of transcription units mapped into each of the regions indicated. The TSS of each transcription unit maps to position 100 on the *x*-axis of each graph. (f) Metagene profiles for conventional NET-Seq and MNase-Seq mapped to the TSS.

As the yeast genome is very compact, the TSS and polyadenylation site (PAS) of all protein-coding regions, plotted as a representative metagene, were used as reference points to assess the distribution and properties of other transcription units in close proximity. The 9542 transcription units were divided into three groups: protein-coding genes, annotated noncoding transcription units (previously identified in the literature) and unannotated noncoding transcription units (identified in this study by sfNET-Seq). Their distributions on both strands near the TSS and PAS of protein-coding genes are displayed as a metagene in Figure 4a.

Seven windows (from A to G) around the protein-coding genes (*n* = 5579) were examined. Windows A–D are on the sense strand relative to the protein-coding genes, while windows E–G are on the antisense strand. Window B shows the TSS distributions for the reference protein-coding gene. Windows D and E focus on the 400 nucleotides upstream or downstream of the TSS/PAS of the coding transcription unit and reveal the TSSs of other protein-coding transcription units, shown by the black lines. The TSSs of the coding transcription units arranged in tandem were centred around +150 from the PAS in window D. There was only a low number of noncoding transcription units initiating within this window. Window E reveals the divergently transcribed protein-coding transcription units, centred about −200 nucleotides upstream of the TSS of the protein-coding metagene. However, in contrast to window D, initiation sites for both annotated (blue line) and unannotated (red line) noncoding transcription units also peaked −200 nucleotides upstream of the TSS. This suggests a chromatin structure or PIC organisation that favours transcription initiation of both coding and noncoding transcription units upstream of protein-coding genes (Murray et al., 2015). As the frequency of all three types of transcription units were similar, it raises interesting questions as to how coding versus noncoding units are determined, given that the noncoding units are twice as abundant as the coding units.

The remaining four windows revealed initiation sites for both annotated and non-annotated noncoding transcription units but not coding transcription units. Within the transcribed region of the protein-coding gene two more windows, C and F, at which noncoding transcripts arise at low frequency, are evident. In window C, the putative TTS are arranged in clusters with a periodicity of 160 nucleotides, which is similar to the periodicity of nucleosome dyads. A similar pattern of TSS is also observed over genes in mammalian cells by CoPRO (Tome et al., 2018).

In window A, upstream of the TSS of the protein-coding metagene on the sense strand in window B, putative initiation sites for noncoding transcription units are evident. The distribution for the unannotated noncoding TSS (red line) has a peak spanning a region between −275 and −190 bps, followed by a trough between −190 and −125 bps. This feature is not seen for the annotated noncoding TSS (blue line). This result indicates that there might be a previously unidentified class of noncoding TSS, which is located at about 240 bp upstream of some protein-coding genes. These transcription units have the potential to mediate transcription interference of the downstream coding region. As their TTS, annotated by sfNET-Seq are highly variable, they are less likely to initiate at a conserved region. Examples of such transcription units discovered by sfNET-Seq are upstream of *YJR147W* (*HMS2*; see Figure 3a) and *YBR162W->A* (*YSY6*; see Figure 3d).

Finally, window G reveals a discrete region for the initiation of both annotated and unannotated noncoding transcription units, 60 nucleotides upstream from the PAS and transcribed on the antisense strand of the protein-coding gene. This suggests a canonical arrangement of nucleosomes and PICs around the PAS, as previously proposed (Murray et al., 2012), similar to that at the TSS. These sites are likely to be the source of the ubiquitous antisense transcripts found throughout the yeast genome (Brooks et al., 2022; Brown et al., 2018; Murray et al., 2015; Pelechano et al., 2013) and are evident in the IGV plots of the sfNET-Seq, particularly at tandemly arranged genes.

### 3.7 Coding and noncoding transcription units have a similar distribution of RNAPII in their promoter proximal regions but distinct genomic features

RNAPII distribution profiles, shown as metagene profiles of NET-Seq data, were plotted for both coding (black) and noncoding (red) transcription units identified by sfNET-Seq (Figure 4b–d). Transcription units were excluded if they have other downstream transcription units within 900–1000 nucleotides. The metagene profiles of transcribed regions downstream of all coding or noncoding TSS are similar showing periodic peaks corresponding approximately to nucleosome positions (Fischl et al., 2017; Zhang & Pugh, 2011) (Figure 4f), although the peaks for the noncoding transcription unit are ‘fuzzier’ and less well positioned. The noncoding transcription units can initiate in very distinct contexts which could explain their fuzziness: near the TSS of coding genes (window E in Figure 4a) or near the PAS of coding genes (window G in Figure 4a). In the latter context, transcription will occur antisense to the protein-coding gene where less well-positioned ‘fuzzy’ nucleosomes are expected (Jiang & Pugh, 2009). After excluding all transcription units initiating within 400nt of an annotated PAS from window E (and thus excluding all the transcription units from window G), the metagene profiles for the remaining transcription units in window E were plotted (Figure 4e). There were similar to those identified genome wide (Figure 4b), suggesting that the fuzzier nucleosome positions associated with noncoding transcription units are not a function of their context.

**Figure 5:**
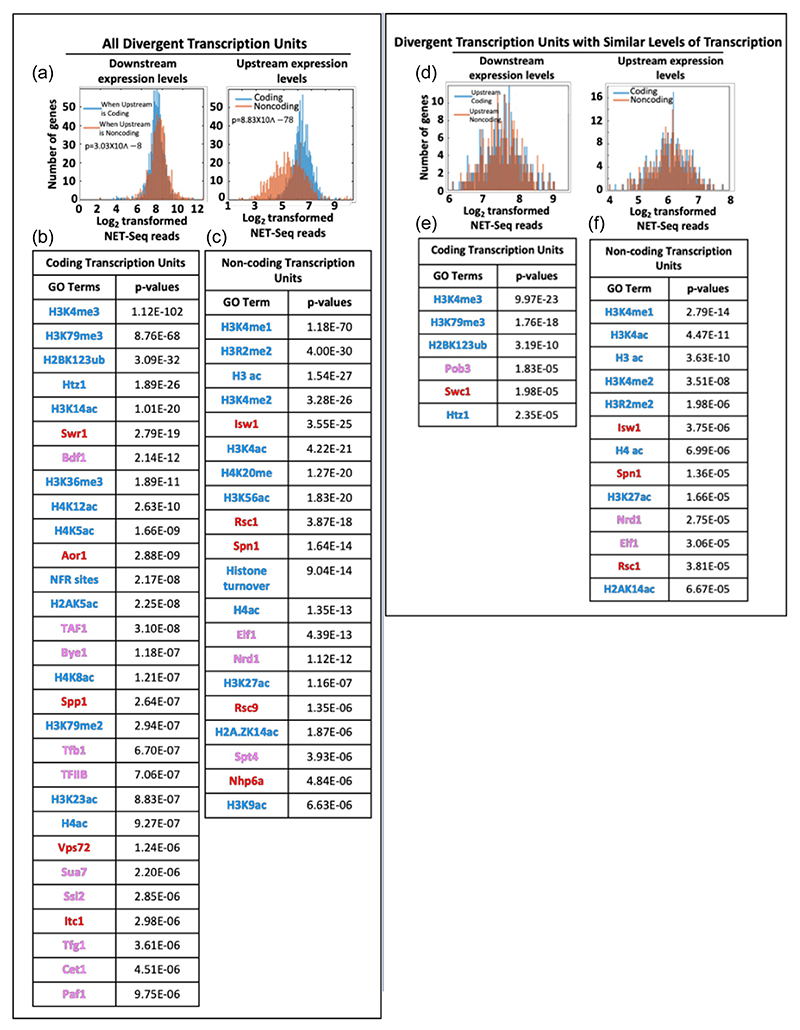
Chromatin features associated with divergent transcription units. In each panel the genomic features (gene ontology GO) associated with the promoter regions of divergent coding and noncoding transcription units are indicated in red, blue and yellow representing chromatin remodelling factors, histone modifications and RNAPII transcription-related factors. A two-sample Kolmogorov–Smirnov test was utilised to examine whether a genomic feature was enriched or depleted around the promoter and early coding regions of coding or noncoding transcription units. The threshold for the significance test was set at the *p* value of 1 × 10^−5^. Any genomic features having their *p* values lower than that were considered significantly enriched for coding or noncoding TSS. This *p* value was equivalent to an *E*-value of 0.015. (a–c) In the left panel all divergent transcription units are shown, including histograms indicating expression levels, which are significantly different when upstream transcription units are either coding or noncoding. (d–f) On the right panel, the same analysis is conducted, except that divergent transcription units with similar levels of coding or noncoding transcription are subject to analysis.

To explore this further, the correlative association between 1453 different genomic features, including various histone modifications and chromatin-associated factors, and the 300nt region downstream of the TSS of these coding and noncoding transcription units were assessed (Figure 5). Each genomic feature has a value to indicate its amount downstream from the TSS for each transcription unit, giving a distribution of values for each genomic feature. A two-sample Kolmogorov–Smirnov test was done to examine whether a genomic feature is enriched or depleted in the promoter region of coding or noncoding transcription units. The threshold for the significance test was set at the *p* value of 1 × 10^−5^. Any genomic feature having lower *p* values was considered significantly enriched for coding or noncoding TSS. This *p* value was equivalent to an *E*-value of 0.015. In other words, it was expected to get 0.015 genomic feature by chance from the analysis.

There are 29 and 20 genomic features significantly enriched at the promoters of divergent coding transcription units and noncoding transcription units, respectively (Figure 5a–c). The coding transcription units are strongly enriched for H3K4me3 and Htz1, together with Bye1 and Spp1, which have been shown to interact with H3K4me3, the Swr1 chaperone complex that incorporates Htz1, Paf1 and many transcription-related factors (Fischl et al., 2017; Kinkelin et al., 2013; Santos Rosa et al., 2003) (Figure 5b). By contrast, at the start of noncoding transcription units, H3K4 is monomethylated, dimethylated or acetylated (not trimethylated) and Htz1 is also acetylated (Figure 5c). Other features identified in a study to explore the effect of high levels of antisense transcription in a 300 nucleotides window downstream from a protein-coding gene promoter are also present around the start of the noncoding transcription unit identified here, including increased histone acetylation, histone turnover, chaperones and Isw1 (Murray et al., 2015) (Figure 5c).

An important control is the ensure that expression levels do not reflect these altered chromatin features. Here, the focus is on divergent promoters. Although levels of RNAPII over coding transcripts when associated with either an upstream coding or noncoding transcription units only show a small difference in NET-Seq reads (*p* = 3.03 × 10^−8^), there are significant differences in NET-Seq signals in the upstream signals, depending on whether they are annotated as coding or noncoding (*p* = 8.8 × 10^−78^) (Figure 5a). Thus, the analysis was repeated with combinations of divergent transcription units where the distribution of NET-seq reads is in a similar range for both the downstream and the upstream transcripts, regardless of whether they were annotated as coding or noncoding (Figure 5d). Although there were fewer transcription units in the analysis, the association with distinct chromatin features remained for the coding or noncoding upstream transcripts (Figure 5e,f). Taken together, this supports the association of coding and noncoding transcription units with different genomic features, but these features do not appear to have a major influence on the promoter proximal distribution of RNAPII on the chromatin template (Figure 4b). Furthermore, this raises the interesting question as to whether chromatin features identified with the 3′ region of yeast protein-coding genes (e.g., H3K4me1) (Soares et al., 2017) are related to the presence of noncoding transcription units in this region and prompted a focus on the PAS region of genes.

### 3.8 In the region flanking the PAS, an upstream noncoding antisense TSS and a downstream coding sense TSS have a strong tendency to co-occur

The genomic features around the divergent coding and noncoding transcription units in windows D and G in Figure 4a, at ~+150 and ~−60 nucleotides with respect to the PAS, were explored. To investigate the potential relationship between the TSS clusters associated with coding and noncoding transcription units in windows D and G, respectively, a single-sided Fisher’s test was performed to examine whether there are co-occurrence relationships between different types of transcription units flanking the PAS of protein-coding genes. As the distributions for annotated and unannotated coding transcription units are similar, they were pooled together as a single group. The null hypothesis for the statistical test is that there is no co-occurrence relationship between the upstream TSS and the downstream TSS. The *p* values for each of the four combinations are summarised in Table 1. All four combinations give low *p* values, which means that there is a general correlation between the upstream and the downstream transcription units. In other words, the transcription units flanking a PAS have a tendency to occur in pairs. Notably, the *p* value for the combination of an upstream noncoding transcription unit and a downstream coding transcription unit is much lower than the other three combinations (*p* = 1.51 × 10^−311^). This strong positive correlation remained when the upstream noncoding transcription units were separated into annotated and nonannotated (Table 1). This is supported by observations from the IGV snapshots which show that the upstream gene in tandemly arranged protein-coding transcription units is often subject to antisense transcription. Both the upstream annotated and the upstream unannotated noncoding TSS strongly correlate with the downstream coding TSS. Although these transcription units co-occur, our previous analyses indicated that they are likely to be initiated from independent transcription pre-initiation complexes (Murray et al., 2012).

### 3.9 The DNA sequences of divergent promoters at the PAS are significantly enriched for the motif of the Reb1-binding site

**Figure 6:**
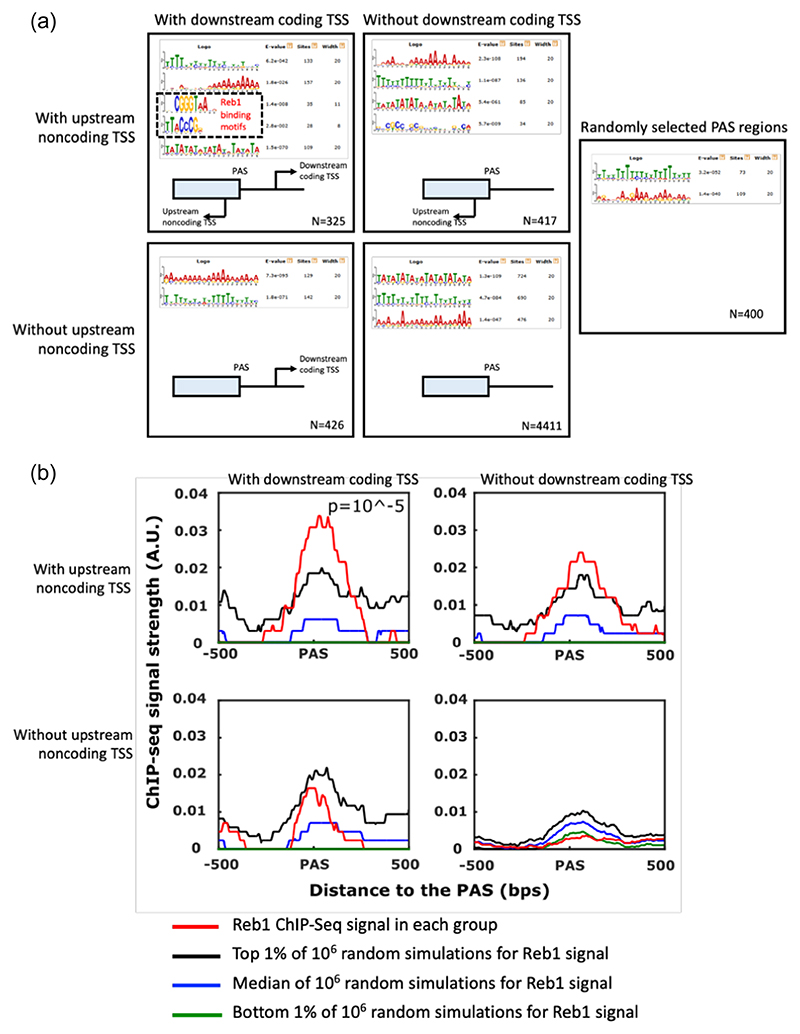
Enriched motifs surrounding the polyadenylation site (PAS) region of protein-coding genes. (a) The regions surrounding the PAS of 5579 protein-coding genes are divided into four groups depending on the presence or absence of an upstream noncoding transcription unit and the presence or absence of a downstream coding transcription unit. A fifth group comprised 400 PAS regions randomly selected from the 5579 protein-coding genes to act as a control. Enriched motifs within each group were discovered using the MEME server and are shown. The number of protein-coding genes in each group is shown in the bottom right-hand corner. (b) The Reb1 ChIP-Seq signals in each of the four types of PAS regions are shown in red. The black, blue and green curves represent the ChIP-Seq signals of the top 1%, median and bottom 1% of one million random simulations. The Reb1 ChIP-Seq signal in the top left-hand panel is significantly enriched compared to the random simulation (*p* = 10^−5^). The ChIP-Seq data were taken from Bosio et al. (2017).

To check whether there are any DNA sequences preferentially associated with the PAS regions having divergent promoters, the MEME (Multiple Em for Motif Elicitation) server (Bailey et al., 2009) was utilised to perform a de novo discovery of enriched DNA sequences for the PAS regions having divergent coding and noncoding transcription units. MEME is based on the Hidden Markov Model (HMM) and does not rely on the prior knowledge of the binding sequences of DNA-associated factors. Although its sensitivity is low, it can discover motifs in an unbiased way. A total of 5579 PAS regions of protein-coding genes were considered, divided into four groups, depending on whether they had an upstream noncoding transcription unit or a downstream coding transcription unit. The MEME was performed on the DNA sequences in each group to discover the enriched DNA motifs relative to all 5579 PAS regions (Figure 6a). To further control for the false discovery of motifs, a fifth group of 400 randomly selected PAS regions from all 5579 was analysed by the MEME server in the same way. Several significantly enriched motifs are found for all five groups. Among them, polyadenosine-rich and polythymidine-rich motifs are found, including in the fifth random control group, which suggests that these motifs are related to the polyadenylation signals or favour the creation of nucleosome-depleted regions as they are associated with distorted DNA histone interactions (Kaplan et al., 2009). The AT alternating motif is not found in the control group, but lacks specificity in the four experimental groups, and thus was not considered for further analysis.

There are three motifs that are only found in individual groups. They are CGGGTAA, TTACCCG and a GC-rich motif. The GC-rich motif is only found in the group with an upstream noncoding transcription unit but without a downstream coding transcription unit. Its median starting position is about 44 bps downstream of the PAS. The motif has a low information content indicated by the heights of the sequence logo, indicating that it is not well conserved and was not considered further. However, the complementary CGGGTAA and TTACCCG motifs, which the YEASTRACT (Yeast Search for Transcriptional Regulators and Consensus Tracking) database (Teixeira et al., 2006) indicate are Reb1-binding sites, are only found in the group containing divergent noncoding and coding transcription units, and their very high information content implies that they are well conserved. The median positions of these two complementary motifs are 28 and 35 bps downstream of the PAS, respectively. Interestingly, both motifs are very close to the middle position between the upstream peak of the noncoding TSS (−60 bps) and the downstream peak of the coding TSS (+140 bps). To conclude, the divergent upstream noncoding and downstream coding transcription units flanking the PAS of a protein-coding gene are significantly associated with the motif of the Reb1 binding site.

### 3.10 The divergent transcription units at the PAS are associated with Reb1 binding

To further examine whether the binding of Reb1 protein was significantly associated with the PAS regions with divergent promoters, the Reb1 ChIP-Seq signals (Bosio et al., 2017) were calculated for each of the four experimental groups (Figure 6b). The metagene of Reb1 ChIP-Seq signals flanking the PAS was plotted for each of the four groups (red lines in Figure 6b). To indicate the significance of the peaks for each group, the same number of PAS regions was randomly selected from all 5579 PAS regions. For each group, this process was repeated 500 times. The median background levels are represented with the blue line, while the top and bottom 1% of the control randomly selected group are indicated by the black and green lines respectively. For the group of PAS regions flanked by divergent upstream noncoding transcription units and downstream coding transcription units, the Reb1 ChIP-Seq signals are much stronger than the top 1% of random simulations. After simulating the background 1,000,000 times, it is estimated that the *p* value for this group is 10^−5^. In this group, the ChIP-Seq signal for Reb1 peaks around 40 bps downstream of the PAS, which is consistent with the positions of Reb1-binding motifs from the motif analysis. For the groups only having a downstream coding transcription unit or only an upstream noncoding transcription unit, their signals are not significantly higher than the background (*p* > 0.01). Interestingly, for the fourth group, its signal is close to the bottom 1% of random simulations, which indicates that the binding of Reb1 is less likely to be associated with a PAS without surrounding transcription units. To sum up, the results suggest that PAS regions with divergent promoters are associated with Reb1 binding.

### 3.11 Reb1 in the context of divergent sense coding and antisense noncoding transcription at the PAS is required to limit levels of antisense noncoding transcription

**Figure 7:**
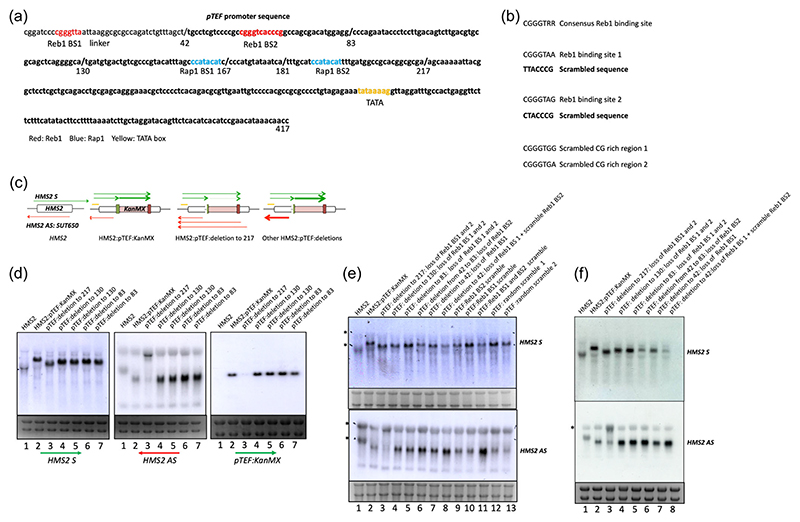
Reb1 functions to limit divergent noncoding transcription. (a) Sequence of *pTEF* (bold), the Reb1 (red) and Rap1 (blue) binding sites and a TATA (yellow) box are indicated, together with the position of each deletion from the 5′ region. The 42nt linker region at the 5′ region of *pTEF* also contains a putative Reb1-binding site (red). (b) Sequences used in scrambling Reb1 BS1 and 2 and the control scrambles in the context of the complete *pTEF* region are indicted. (c) The direction, approximate levels and length of the transcripts detected are indicted in the schematics. (d–f) Northern blots and strand-specific probes (see Figure S3A) were used to monitor the size and levels of transcripts produced from the WT *HMS2* locus, *HMS2* with *pTEF->KanMX* insert at 650 bp, and the same construct with engineered Reb1 and Rap1 binding sites in *pTEF*. The precise sequences engineered are indicated above each track of the Northern blots. * marks the position of the rRNA on the blots and the degree of cross-hybridisation with the *HMS2* AS probe.

Reb1 is a transcription factor that is proposed to act as a roadblock to terminate the transcription of RNAPII (Candelli et al., 2018; Colin et al., 2014) to create a nucleosome-depleted region by recruiting chromatin remodellers to promoters (Hartley & Madhani, 2009; Krietenstein et al., 2016; Rossi et al., 2021) and to limit divergent noncoding transcription (Challal et al., 2018; Wu et al., 2022). To investigate how Reb1 functions in the context of divergent noncoding:coding transcription units, a previously created hybrid divergent transcription unit, made by inserting the p*TEF->KanMX*-t*TEF* cassette into the *HMS2* locus so that the two protein-coding genes flank pairs of Reb1 and Rap1 binding sites in the *TEF* promoter, was subject to mutational analysis (Figure 7 and Figure S3) (Nguyen et al., 2014). Sequential deletions throughout the 417nt of *pTEF* (Figure 7a), or scrambling sequences to maintain spacing and GC content (Figure 7b), were used to monitor how individual transcription factor-binding sites affect the levels and integrity of transcripts (Figure 7c). Strand-specific probes were used to detect the transcripts (Figure S3A). Transcripts initiated in four regions; sense transcripts initiated from the *HMS2* promoter and the *TEF* promoter while antisense transcripts initiated from the *TEF* promoter and from the *HMS2* antisense promoter (Figure 7c) (Nguyen et al., 2014). For all transcripts, their termination site varied, often leading to readthrough transcripts. The *HMS2* sense readthrough transcript transcribed over *HMS2* into *pTEF:KanMX*, the *HMS2* antisense transcript (*SUT650*) and the divergent noncoding transcript initiating from p*TEF*, transcribed on the antisense strand of *HMS2*, displayed nuclear exosome (Rrp6)-sensitivity (Figure S3B).

Deletion of 217 bp, containing both pairs of Reb1 and Rap1 binding sites in *pTEF*, leaving 200 bp including the TATA region intact (Figure 7a), reduces levels of the both the *KanMX* sense transcript and the divergent antisense transcript, although the transcript from the *HMS2* promoter is not affected (Figure 7d, lane 3). When all the Rap1 and Reb1 transcription factor binding sites are ablated in the 217 bp deletion, a new antisense transcript (>3bk), initiating in the same region as the natural *HMS2* antisense transcript (*SUT650*), at the 3′ end of *HMS2* and extending antisense to the *pTEF:KanMX:t-TEF* cassette into the *HMS2* promoter, is produced (Figure 7c,d, lane 3). A single Rap1-binding site (BS2), remaining after deletion of 167 or 181 bp of *pTEF* from the 5′ region, is sufficient to maintain divergent transcription from *pTEF:KanMX* and to block antisense transcription from the 3′ end of *HMS2* (Figure S3C, lanes 3–5). It is not clear if this blocking effect is related to high level of transcription of the *KanMX* cassette or whether it is a specific function of Rap1, but this was not investigated further.

By contrast, the strains lacking both Reb1-binding sites, for example, by removing 83 or 130 nucleotides from the 5′ region of the *pTEF*, led to an increase in the levels of the *HMS2* antisense transcript, but no change in levels of readthrough transcripts from the *HMS2* promoter or in the *KanMX* sense transcript (Figure 7a,d, lanes 4–7, f, lanes 4–5). To demonstrate that this was a function of the Reb1-binding sites, not other sequences, each of the Reb1-binding sites was scrambled individually or together (Figure 7b,e, lanes 8–11). As a control, two other regions close to the Reb1 BS1 and BS2 in *pTEF* with similar GC content were scrambled (Figure 7b,e, lanes 12–13). The constructs lacking one or both Reb1-binding sites lead to an increase in the antisense transcript (Figure 7e, lanes 6–7, f, lanes 6–8), particularly the strain which both Reb1-binding sites scrambled in the context of p*TEF* (Figure 7e, lane 11). This strongly supports a role for Reb1 in limiting levels of noncoding antisense transcription in this particular context.

For some constructs in which the consensus downstream Reb1-binding site (BS2) is deleted or scrambled in the context of the whole p*TEF* promoter, but not lost as a result of sequential deletion, levels of the readthrough transcript initiating at the *HMS2* promoter are also reduced, although their integrity is maintained (Figure 7e, lanes 6, 8, 9, 11, f, lanes 6 and 8). This raises the interesting possibility that the influence of antisense transcription on the *HMS2* promoter is converted from neutral to repressive, consistent with the idea that properties of genetic sequences change as their environment is altered (Brooks et al., 2022).

## 4 Discussion

This study describes size fractionated NET-Seq (sfNET-Seq), an adaptation of the native elongating transcript (NET-Seq) method, for unambiguous determination of transcription units in the yeast genome. From this, a high-resolution architectural map of RNA polymerase II transcription units for the budding yeast genome has been constructed. sfNET-Seq maps transcription units by the distinctive pattern among several libraries constructed with nascent transcripts of different size ranges rather than merely finding peaks higher than a background threshold in a single library as in no-tag subtracted NET-Seq (Fischl et al., 2017). PRO-CAP also relies on finding peaks at single nucleotides above an experimental threshold to map TSS and it can be hard to distinguish the TSS for many bone fide transcription units in the data sets. sfNET-Seq offers the advantage of being able to assign a TSS based on the distribution of reads expected for a transcription unit, with the sensitivity to distinguish these transcription units from background or threshold signals. The pattern recognition algorithm used to define the TSS of a transcription unit is expressed as a correlation coefficient, allowing lowly expressed transcription units to be detected with equally high sensitivity and confidence. One potential disadvantage with sfNET-Seq is that co-transcriptional cleavage events, such as splicing at the intron–exon boundaries and the cleavage at the PAS could be misannotated as start sites for transcription. However, as these are on the same strand as the main coding transcript, they can be removed bioinformatically and are unlikely to be a major issue because the genome of budding yeast does not have many genes containing introns. The presence of introns in mammalian genomes may limit some applications of sfNET-Seq but could be overcome by size fractionating over a finer size range.

As the starting material of sfNET-Seq is nascent RNA associated with RNA polymerase II, the identification of TSS and transcription units is less influenced by the nuclear surveillance and cytoplasmic degradation pathways. The noncoding transcription units, including NUTs, SUTs, CUTs and XUTs, can be detected in glucose-grown cultures using sfNET-Seq and importantly, without mutating the cellular RNA degradation systems, avoiding potential artefacts due to stress responses (O'Duibhir et al., 2014). The 3113 newly identified unannotated transcription units are likely to be noncoding, and generally have low transcription levels in the log phase batch culture conditions. The position and number of these noncoding transcripts support their potential to influence gene transcription and expression. Transcripts initiating on the same strand upstream of protein-coding genes have the potential to mediate transcriptional interference of the downstream gene. Perhaps the best characterised of these is the highly expressed *SRG1* transcription unit, mediating transcriptional interference of the downstream *SER3* coding region (Martens et al., 2004) but sfNET-Seq reveals many more unannotated transcription units upstream of coding genes and transcribed over their promoters into the gene body. Such transcripts contribute to the high number of transcript isoforms in the yeast transcriptome (Pelechano et al., 2013). One such low abundance unannotated transcript, initiating upstream of *HMS2* and extending over 2 kb through the 3′UTR, is characterised here to validate the potential of sfNET-Seq to discover new transcription units. Such isoforms are often low abundance transcripts, are found in many genomes and may well determine the site of 3′ end formation (Alfonso-Gonzalez et al., 2023; Nguyen et al., 2014; Thomas et al., 2020; Wang et al., 2020).

Divergent transcripts on the opposite strand to the protein-coding gene are common across species and often arise from divergent promoters with a shared NDR and two regions for pre-initiation complex (PIC) formation. In yeast, the distinct PICs are proposed to be insulated from one another by nucleosomes that are organised by transcription factors such as Abf1 and Reb1 (Candelli et al., 2018; Krietenstein et al., 2016). A high-resolution analysis reveals that 81% of yeast genes have promoters found within a short nucleosome-depleted region (NDR), an organisation that favours constitutive low-level expression (Rossi et al., 2021). In support of the idea that divergent transcription arises when nucleosome-depleted regions are formed by DNA-binding factors, we found that targeting dCAS9 to chromatin gives rise to divergent noncoding transcription flanking the dCAS9-binding site (Howe et al., 2017).

In yeast, the distance between the two divergent core promoters at the 5′ region of protein-coding genes is similar (approx. 200nt) regardless of whether the divergent transcription unit encodes an pre-mRNA or a noncoding transcript. In yeast these noncoding transcripts are known as upstream antisense transcripts and in mammalian cells as PROMPTs (promoter upstream transcripts). PROMPTs tend to become exosome targets when they are not spliced, and dependent on their proximity and degree of overlap with other transcription units (Chen et al., 2016). Convergent overlapping transcription units which are common in mammalian cells also give rise to PROMPTs, antisense to other transcription units which also showed differential sensitivity to the nuclear exosome (Chen et al., 2016) but there was little evidence for equivalent transcripts in the yeast genome in which genes are close to each other but tend not to overlap.

Much more common in the compact yeast genome are upstream antisense transcription units arising within or close to the 3′ end of protein coding genes and often subject to degradation by one or more of the nuclear and/or cytoplasmic degradation machineries. Much of this upstream antisense transcription is coregulated with the downstream protein coding gene (Nguyen et al., 2014) and as shown here, Reb1 is required to coordinate this. This transcription extends well into the coding region of the upstream gene and has a marked influence on the sense transcript fate through its effects on the local chromatin structure (Brown et al., 2018; Murray & Mellor, 2016; Murray et al., 2015). We confirm the presence of a large number of noncoding nascent transcripts associated with a distinct set of chromatin features and with regulatory potential, including increasing, decreasing or having no apparent effect on neighbouring nascent transcripts (Brown et al., 2018; Murray & Mellor, 2016; Murray et al., 2015; Nguyen et al., 2014; Pelechano et al., 2014). Indeed, each act of transcription leaves a novel set of chromatin modifications that can influence how a region is transcribed and the fate of the encoded transcript (Brown et al., 2018).

When positioned close to the polyadenylation site between two tandemly arranged genes, Reb1 is required to limited levels of antisense transcription from the divergent promoter, although there is no effect on levels of the downstream sense transcript, consistent with the idea that Reb1 functions normally to uncouple divergent transcription (Rossi et al., 2021). By contrast, a single Rap1-binding site is sufficient to maintain levels of both divergent transcripts, supporting a role in creating a permissive chromatin environment for divergent transcription as proposed (Badis et al., 2008; Kubik et al., 2015; van Bakel et al., 2013; Yan et al., 2018). In conclusion, sfNET-Seq has uncovered a new architecture of transcription units around yeast genes and implicated transcription factors such as Rap1 and Reb1 in determining the functional consequences of such pervasive transcription.

## Supplementary Material

Additional supporting information can be found online in the Supporting Information section at the end of this article.

Scripts for bioinformatics analysis of sfNET-Seq

## Figures and Tables

**Table 1:** Correlations between divergent transcription units flanking the PAS.

	Downstream coding	Downstream noncoding	Downstream annotated noncoding	Downstream unannotated noncoding
Upstream coding	5.11 × 10^-05^	1.32 × 10^-08^	1.57 × 10^-06^	0.000907585
Upstream noncoding	**1.51**× **10**^-**311**^	9.15 × 10^-12^	-	-
Upstream annotated noncoding	**4.67**× **10**^-**159**^	-	0.140176503	0.000390385
Upstream unannotated noncoding	**4.09**× **10**^-**96**^	-	0.01240602	3.38 × 10^-06^

*Note*: Correlations between upstream antisense (relative to the PAS) coding/noncoding transcription units and downstream (relative to the PAS) coding/noncoding transcription units are tested by single-sided Fisher’s tests. The noncoding transcription units are further divided into the annotated and unannotated noncoding transcription units. Combinations with a highly significantly positive correlation are highlighted in bold.

## Data Availability

The data underlying this article are available in GEO at https://www.ncbi.nlm.nih.gov/geo/query/acc.cgi?acc=GSE246153.

## Take-away

- Size fractionated NET-Seq maps nascent transcripts in *Saccharomyces cerevisiae*.
- sfNET-Seq discovers transcription units independent of expression levels.
- sfNET-Seq resolves overlapping and lowly expressed transcription units
- sfNET-Seq reveals divergent promoters at the 5′ and 3′ ends of coding genes.
- Between tandem genes, Reb1 limits upstream antisense noncoding transcription.
