# Supplementary material for: Size fractionated NET-Seq reveals a conserved architecture of transcription units around yeast genes
Source: Yeast. Author manuscript; Available in PMC 2026 Feb 19. (PMC7618759; doi:10.1002/yea.3931)
Supplement: Scripts for bioinformatics analysis of sfNET-Seq [file EMS211156-supplement-Scripts_for_bioinformatics_analysis_of_sfNET_Seq.pdf]

Scripts for bioinformatic analysis of sfNET-Seq libraries

```
load('size_fractionated_NETseq.mat')
%smoothing the data of size fractionated NETseq
d1 =
designfilt('lowpassiir','FilterOrder',12,'HalfPowerFrequency',0.05,'DesignMethod','butter');
for i=1:5
  size_fractionated_NET_seq{2,i}=cell(16,1);
  for j=1:16

size_fractionated_NET_seq{2,i}{j,1}(:,1)=filtfilt(d1,size_fractionated_NET_seq{1,i}{j,1}(:,1));

size_fractionated_NET_seq{2,i}{j,1}(:,2)=filtfilt(d1,size_fractionated_NET_seq{1,i}{j,1}(:,2));
  end
end

%making the reference pattern, require the function of 'YMC_metagene'.
%reference cerevisiae_annotation is based on TIF-seq
for i=1:5
[S(i,1:2000),~]=YMC_metagene(size_fractionated_NET_seq{1,i},cerevisiae_annotation,1,180
0,5,1:2000,'k',0,1,200);
hold on;
end
Standard=[S(1,100:300),S(2,200:400),S(3,200:400)];
Standard=Standard*10000;
Standard=Standard';


%calculate the correlation coefficient on each nucleotide position
R=cell(16,1);
for chrom=1:16
    length=genome_file(chrom,2);
    R{chrom,1}=zeros(length,2);
    Temp2=zeros(603,1);
    for Start=102:length-599
      Temp2(1:201,1)=size_fractionated_NET_seq{2,1}{chrom,1}(Start-101:Start+99,1);
      Temp2(202:402,1)=size_fractionated_NET_seq{2,2}{chrom,1}(Start-1:Start+199,1);
      Temp2(403:603,1)=size_fractionated_NET_seq{2,3}{chrom,1}(Start-1:Start+199,1);
      R{chrom,1}(Start,1)=corr(Temp2,Standard);
    end
    for Start=length-101:-1:600
      Temp2(1:201,1)=size_fractionated_NET_seq{2,1}{chrom,1}(Start+101:-1:Start-99,2);
      Temp2(202:402,1)=size_fractionated_NET_seq{2,2}{chrom,1}(Start+1:-1:Start-199,2);
      Temp2(403:603,1)=size_fractionated_NET_seq{2,3}{chrom,1}(Start+1:-1:Start-199,2);
      R{chrom,1}(Start,2)=corr(Temp2,Standard);
    end
end
```

```
%Find peaks on the plot of correlation coefficient
peaks=cell(16,2);
for i=1:16
[~,peaks{i,1}]=findpeaks(R{i,1}(:,1),'MinPeakHeight',0.34,'MinPeakDistance',50);
[~,peaks{i,2}]=findpeaks(R{i,1}(:,2),'MinPeakHeight',0.34,'MinPeakDistance',50);
end

%Only select the positions with at least 26 reads in the window from 100bp
%upstream to 200bps downstream

for chrom=1:16
temp=size(peaks{chrom,1});
temp1=size(peaks{chrom,2});
for j=1:temp(1,1)
Start=peaks{chrom,1}(j,1);
Temp2=sum(size_fractionated_NET_seq{1,1}{chrom,1}(Start-101:Start+99,1));
Temp3=sum(size_fractionated_NET_seq{1,2}{chrom,1}(Start-1:Start+199,1));
Temp4=sum(size_fractionated_NET_seq{1,3}{chrom,1}(Start-1:Start+199,1));
peaks{chrom,1}(j,2)=sum(Temp2+Temp3+Temp4);
end
for j=1:temp1(1,1)
Start=peaks{chrom,2}(j,1);
Temp2=sum(size_fractionated_NET_seq{1,1}{chrom,1}(Start+101:-1:Start-99,2));
Temp3=sum(size_fractionated_NET_seq{1,2}{chrom,1}(Start+1:-1:Start-199,2));
Temp4=sum(size_fractionated_NET_seq{1,3}{chrom,1}(Start+1:-1:Start-199,2));
peaks{chrom,2}(j,2)=sum(Temp2+Temp3+Temp4);
end
end
for i=1:16
peaks{i,1}(peaks{i,1}(:,2)<26,:)=[];
peaks{i,2}(peaks{i,2}(:,2)<26,:)=[];
end

%assign the correlation coefficient to each TSS
for chrom=1:16
   for j=1:length(peaks{chrom,1}(:,1))
      position_plus=peaks{chrom,1}(j,1);
      peaks{chrom,1}(j,3)=R{chrom,1}(position_plus,1);
   end
   for j=1:length(peaks{chrom,2}(:,1))
      position_minus=peaks{chrom,2}(j,1);
      peaks{chrom,2}(j,3)=R{chrom,1}(position_minus,2);
   end
end
```
